# Time series clustering of mRNA and lncRNA expression during osteogenic differentiation of periodontal ligament stem cells

**DOI:** 10.7717/peerj.5214

**Published:** 2018-07-16

**Authors:** Yunfei Zheng, Xiaobei Li, Yiping Huang, Lingfei Jia, Weiran Li

**Affiliations:** 1 Department of Orthodontics, Peking University School and Hospital of Stomatology, Beijing, China; 2 Department of Oral and Maxillofacial Surgery, Peking University School and Hospital of Stomatology, Beijing, China; 3 Central Laboratory, Peking University School and Hospital of Stomatology, Beijing, China

**Keywords:** lncRNAs, Osteoblast, PDLSCs, STEM

## Abstract

**Background:**

Long noncoding RNAs (lncRNAs) are regulatory molecules that participate in biological processes such as stem cell differentiation. Periodontal ligament stem cells (PDLSCs) exhibit great potential for the regeneration of periodontal tissue and the formation of new bone. However, although several lncRNAs have been found to be involved in the osteogenic differentiation of PDLSCs, the temporal transcriptomic landscapes of mRNAs and lncRNAs need to be mapped to obtain a complete picture of osteoblast differentiation. In this study, we aimed to characterize the time-course expression patterns of lncRNAs during the osteogenic differentiation of PDLSCs and to identify the lncRNAs that are related to osteoblastic differentiation.

**Methods:**

We cultured PDLSCs in an osteogenic medium for 3, 7, or 14 days. We then used RNA sequencing (RNA-seq) to analyze the expression of the coding and non-coding transcripts in the PDLSCs during osteogenic differentiation. We also utilized short time-series expression miner (STEM) to describe the temporal patterns of the mRNAs and lncRNAs. We then performed Gene Ontology and Kyoto Encyclopedia of Genes and Genomes analyses to assess the biological relevance of genes in each profile, and used quantitative real-time PCR (qRT-PCR) to validate the differentially expressed mRNAs and lncRNAs that were associated with osteoblast differentiation. Lastly, we performed a knock down of two lncRNAs, MEG8, and MIR22HG, and evaluated the expression of osteogenic markers.

**Results:**

When PDLSCs were differentiated to osteoblasts, mRNAs associated with bone remodeling, cell differentiation, and cell apoptosis were upregulated while genes associated with cell proliferation were downregulated. lncRNAs showed stage-specific expression, and more than 200 lncRNAs were differentially expressed between the undifferentiated and osteogenically differentiated PDLSCs. Using STEM, we identified 25 temporal gene expression profiles, among which 14 mRNA and eight lncRNA profiles were statistically significant. We found that genes in pattern 12 were associated with osteoblast differentiation. The expression patterns of osteogenic mRNAs (COL6A1, VCAN, RRBP1, and CREB3L1) and lncRNAs (MEG8 and MIR22HG) were consistent between the qRT-PCR and RNA-seq results. Moreover, the knockdown of MEG8 and MIR22HG significantly decreased the expression of osteogenic markers (runt-related transcription factor 2 and osteocalcin).

**Discussion:**

During the osteogenic differentiation of PDLSCs, both mRNAs and lncRNAs showed stage-specific expression. lncRNAs MEG8 and MIR22HG showed a high correlation with osteoblastogenesis. Our results can be used to gain a more comprehensive understanding of the molecular events regulating osteoblast differentiation and the identification of functional lncRNAs in PDLSCs.

## Introduction

The periodontal ligament (PDL) bridges the space between the root cement and the surrounding alveolar bone and is essential for the preservation and function of teeth. Infectious diseases such as periodontitis as well as excessive mechanical force during orthodontic treatment lead to the progressive destruction of periodontal bone (Khan et al., 2015; Matsumoto, Sringkarnboriboon & Ono, 2017). Periodontal tissue regeneration is the ultimate goal of periodontal therapy, of which bone regeneration is a crucial part (Ramseier et al., 2012). The progenitor cells isolated from the PDL, namely periodontal ligament stem cells (PDLSCs), retain stem cell-like properties and can be differentiated into different lineages (Seo et al., 2004). PDLSCs also exhibit great potential for the regeneration of periodontal tissues, including the formation of new bone (Liu et al., 2008). Although efforts have been made in the past to promote the osteogenic differentiation of PDLSCs, the molecular mechanism regulating differentiation potency is still not fully understood.

Emerging evidence shows that long noncoding RNAs (lncRNAs) act as key regulators of biological and pathological processes (Huynh et al., 2017; Lin, Wu & Shan, 2012), and they also play an important role in stem cell self-renewal and multidirectional differentiation (Hutchins & Pei, 2015; Perry & Ulitsky, 2016; Xu et al., 2016). Studies suggest that lncRNAs are involved in the differentiation of bone marrow-derived mesenchymal stem cells (MSCs) undergoing osteogenic differentiation, such as lncRNA-MEG3, Malat1, H19, MIR31HG, DANCR, and HoxA-AS3 (Huang et al., 2015; Jin et al., 2016; Tang, Gong & Sun, 2018; Xiao et al., 2017; Zhu et al., 2016; Zhuang et al., 2015; Zuo et al., 2013). But although bone marrow-derived MSCs and PDLSCs have similar differentiation potential, these two kinds of cells have different tissue origins and the molecular mechanisms governing their differentiation are cell specific (Li et al., 2014; Zhang et al., 2014). Thus, the regulatory role of lncRNAs in PDLSC differentiation requires further investigation.

As a preferred high-throughput technique for genome-wide gene expression quantification, the RNA deep-sequencing method RNA sequencing (RNA-seq) allows for sensitive, comprehensive, and accurate profiling of the transcriptome (Nikiforova et al., 2016). During multistage osteoblastogenesis, the timing of gene expression is important and precisely controlled (Hayami, Kapila & Kapila, 2011; Li et al., 2008; Kawasaki et al., 2010). Thus, time-course data can help identify important genes that regulate osteoblast differentiation. The bioinformatics method of short time-series expression miner (STEM) analysis can be used to determine statistically significant time-dependent gene expression profiles that closely resemble the biology of cells (Ernst, Nau & Bar-Joseph, 2005). The STEM algorithm organizes all genes into sets with predefined patterns and identifies genes with similar profiles over a short time series. In this study, we used STEM analysis to reveal a collection of lncRNAs whose expressions might correlate with osteoblastic differentiation of PDLSCs.

## Materials and Methods

### Cell isolation and culture

We isolated human PDLSCs using the process described previously (Seo et al., 2004). Briefly, we collected PDL tissue from the middle third root of healthy premolars, cut it into pieces, and digested it in equal volumes of type I collagenase and dispase. Next, we cultured the isolated cells using α-Modified Eagle Medium (α-MEM) supplemented with 10% fetal bovine serum and 1% penicillin/streptomycin (Gibco, Grand Island, NY, USA). We passaged the cells using 0.25% trypsin and further expanded them to passage 4 before they were used in the experiments. For osteogenic induction, we cultured PDLSCs in α-MEM supplemented with 10% FBS, 100 nM dexamethasone, 200 μM L-ascorbic acid, and 10 mM β-glycerophosphate. We changed the medium every 2 days and harvested the cells at the indicated time points. The study protocol was approved by the Ethics Committee of Peking University School of Stomatology (PKUSSIRB-2011007).

### RNA sequencing

We performed sequencing of the mRNA and lncRNA as described previously (Zheng et al., 2016; Zheng & Jia, 2016). Briefly, we extracted the RNA of the PDLSCs using the Trizol reagent (Invitrogen Life Technologies, Carlsbad, CA, USA). After digestion with DNase, we depleted rRNA using a Ribo-Zero magnetic kit and prepared a library according to the manufacturer’s instructions (Epicentre Biotechnologies, Madison, WI, USA). We performed paired-end sequencing using the Illumina Hiseq X Ten. After removing low-quality reads, we trimmed the raw reads and mapped them to the human genome (hg19) using TopHat2. We used HTseq to count the genes and calculated the reads per kilobase transcriptome per million mapped reads (RPKM) to evaluate the gene expression level. We used the EB-Seq package for the differential gene expression analysis among PDLSCs at each time point. We defined the differentially expressed genes (DEGs) based on fold changes greater than or equal to 2.0 and a false discovery rate of less than 0.05. We performed Gene Ontology (GO) and Kyoto Encyclopedia of Genes and Genomes (KEGG) analyses using the Database for Annotation Visualization and Integrated Discovery. Raw data of the performed RNA-seq have been recorded in the GEO public database (accession number: GSE99958).

### STEM analysis and Gene Set Enrichment Analysis

We used the STEM (Ernst, Nau & Bar-Joseph, 2005) clustering algorithm to identify temporal gene expression profiles during the osteogenic differentiation of PDLSCs. Representative temporal expression profiles were first defined as model profiles, which were independent from the sequencing data. The values of the gene expression were transformed to log ratios relative to the expression at Day 0 (D0). Then each gene was assigned to the filtering criteria of the model profiles, and the correlation coefficient was determined. We performed standard hypothesis testing using the true ordering of time points, and determined the *p*-value using the number of genes assigned to the model profile and the expected number of assigned genes (adjusted *p*-value, 0.05 by Bonferroni correction). The boxes of figures were colored if the profiles were statistically significant. We also performed the Gene Set Enrichment Analysis (GSEA) to identify the GO biological processes involving genes with significant profiles as previously described (Subramanian et al., 2007).

### Alkaline phosphatase staining

We undertook a histochemical analysis of alkaline phosphatase (ALP) activity as previously described (Huang et al., 2015). For the ALP staining, we seeded cells in 24-well plates and cultured them in an osteogenic medium for 3, 7, or 14 days. Subsequently, we fixed the cells in 4% paraformaldehyde for 30 min. After washing them with PBS, we incubated the cells for 20 min at 37 °C in an alkaline solution according to the directions for the NBT/BCIP staining kit (CoWin Biotech, Beijing, China). We took images of PDLSCs using a Zeiss system.

### Mineralization assay

To visualize the mineral deposition in the extracellular matrix (ECM), we cultured the PDLSCs in an osteogenic medium for 3, 7, or 14 days. We then fixed the cells in 4% paraformaldehyde and stained them with 0.1% Alizarin Red S (ARS; Sigma-Aldrich, St. Louis, MO, USA) for 20 min at room temperature. We took images of this samples using a Zeiss system.

### Quantitative realtime PCR

We extracted total RNA using the Trizol reagent and carried out quantitative real-time PCR (qRT-PCR) in triplicate using the Power SYBR Green® PCR Mastermix (Applied Biosystems, Foster City, CA, USA) as previously described (Zheng et al., 2016). The mean expression values were calculated relative to the mean expression level of the housekeeping gene GAPDH. The primers that we used are listed in Table 1. We conducted a statistical analysis using one-way ANOVA, followed by Student–Newman–Keuls post hoc tests using SPSS 16.0 software.

**Table 1:** The qRT-PCR primers used in this study.

	Forward	Reverse
GAPDH	5′-CGACAGTCAGCCGCATCTT-3′	5′-CCAATACGACCAAATCCGTTG-3′
RUNX2	5′-ACTACCAGCCACCGAGACCA-3′	5′-ACTGCTTGCAGCCTTAAATGACTCT-3′
OCN	5′-ACCCTGACCCATCTCAGAAGCA-3′	5′-CTTGGAAGGGTCTGTGGGGCTA-3′
COL6A1	5′-ACAGTGACGAGGTGGAGATCA-3′	5′-GATAGCGCAGTCGGTGTAGG-3′
VCAN	5′-GTAACCCATGCGCTACATAAAGT-3′	5′-GGCAAAGTAGGCATCGTTGAAA-3′
RRBP1	5′-TACGACACTCAAACCTTGGGG-3′	5′-GGTTGGCTAGGGCTTCTTCATA-3′
CREB3L1	5′-CCTCCCGAAGCCTCCTATTCT-3′	5′-GGGGTTGATTTCCCAGCCA-3′
MEG8	5′-CCTGGGCTGGCAGAACATC-3′	5′-CCACCGTCGCTACAGGATGA-3′
MIR22HG	5′-TGTATCTTGTCCTCCGCTTGTG-3′	5′-GCCTATGAGTCTATCCCCTGC-3′

### siRNA knock down assay

Periodontal ligament stem cells were transfected with siRNA to knock down the expression of MEG8 and MIR22HG. Cells in six-well plates were transfected with 10 µl siRNA, 250 µl Opti-MEM medium, and three µl Lipofectamine® RNAiMAX (Life Technologies, Carlsbad, CA, USA). Total RNA was extracted 24 h after transfection and the expression levels of runt-related transcription factor 2 (RUNX2) and osteocalcin (OCN) were examined.

## Results

### Summary of the RNA-seq data

**Figure 1:**
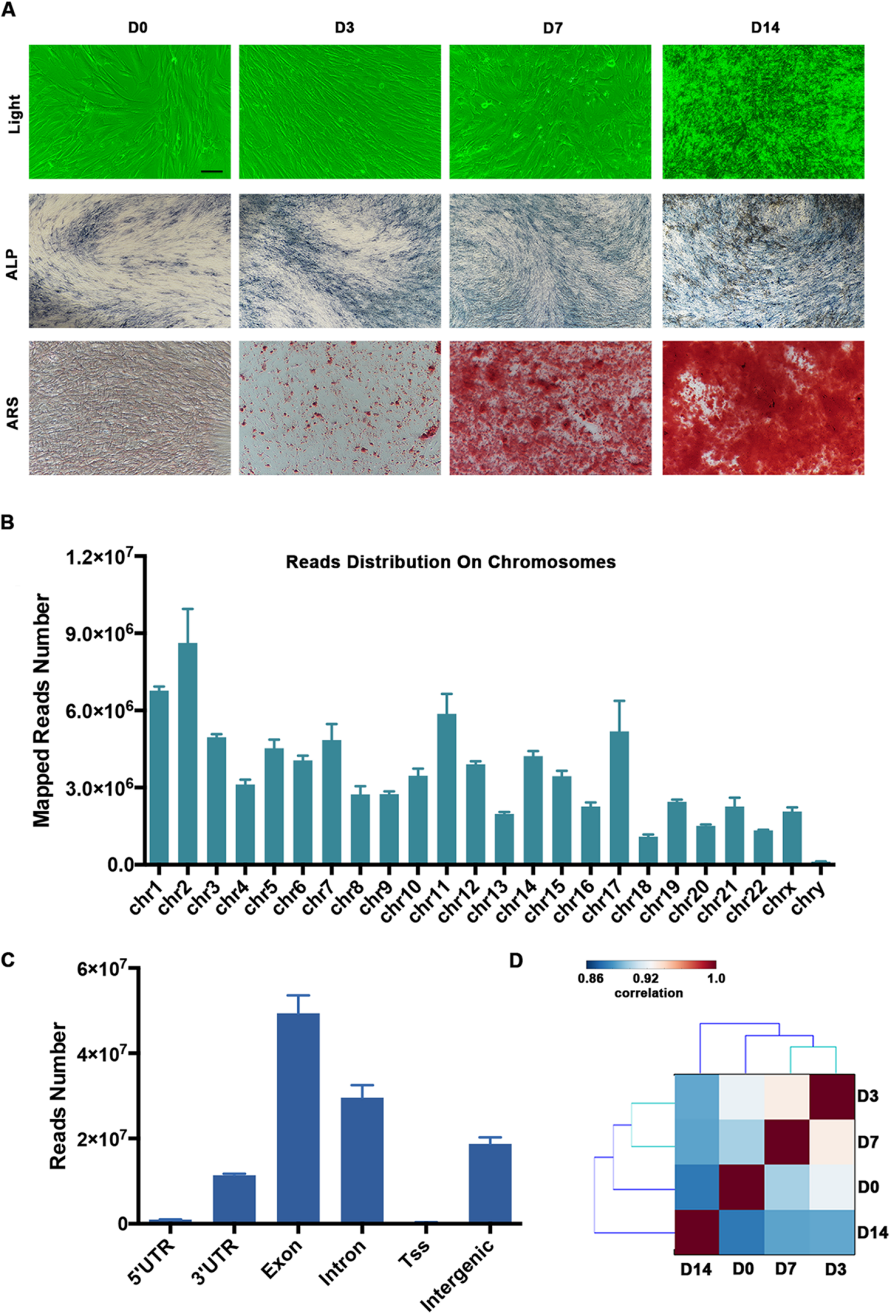
Summary of our RNA-seq data. (A) Representative images showing the morphology and osteogenesis of isolated PDLSCs. ALP and ARS staining of PDLSCs was used to confirm the formation of calcified nodules. Scale bar: 50 μm. (B) The distribution of mapped reads on the chromosomes. (C) The mapped reads were classified according to the gene structure, and more than 90% were localized to the exon and intron regions. (D) Pairwise correlation analyses of samples showed that the PDLSCs on D3 and D7 were highly correlated. The pairwise correlation was performed by multiple Pearson analyses. The outputs were hierarchically clustered based on dissimilarity measures. The outputs are given in a correlation matrix, and the color of the square indicates the magnitude of the correlation. The color bar refers to the correlation coefficient. Blue colors have a low correlation coefficient, and red colors have a high correlation coefficient.

The human PDLSCs showed osteogenic differentiation potential when they were cultured in the osteogenic medium. The calcified nodules and ARS staining of the PDLSCs were significantly increased on Days 7 and 14 (D7 and D14, respectively; Fig. 1A). Using the RNA-seq technique, we determined the mRNA and lncRNA expression profiles of the PDLSCs during osteogenic differentiation, which provided insight into the molecular mechanisms underlying osteoblast differentiation. We generated paired-end libraries and sequenced more than 2 × 10^8^ reads per sample. We filtered the raw reads (Fig. S1) and mapped them to the hg19 version of the human genome. Our analysis of the gene structure, as well as their distribution on the chromosomes of the mapped reads, is shown in Figs. 1B and 1C. Generally, the mapping numbers per chromosome correlated broadly with chromosome length (Fig. 1B). Chromosome Y received a relatively low number of read mappings. The majority of reads were localized in the exon and intron regions. Moreover, the intergenic regions produced a great number of transcripts (Fig. 1C). Pairwise correlation analyses using the gene-level expression data demonstrated that there was a high degree of similarity between the PDLSCs on Days 3 (D3) and D7 (Fig. 1D).

### Characterization of the mRNA transcriptome of PDLSCs

**Figure 2:**
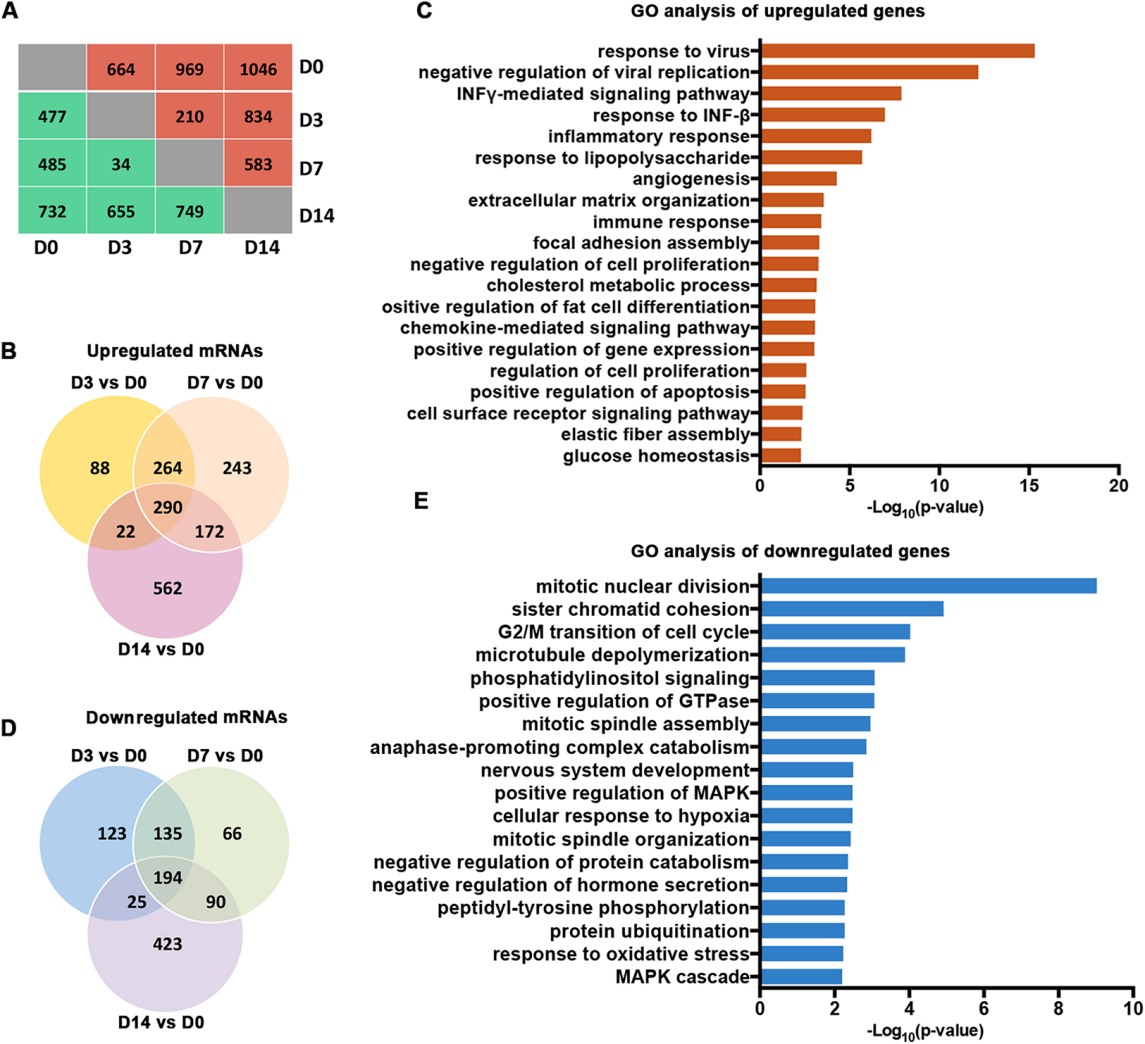
Characterization of the mRNA transcriptomes of PDLSCs. (A) The number of DEGs across samples. The red brackets indicate upregulated genes and the blue ones indicate downregulated genes. (B) Venn diagram of upregulated genes that were significantly differentially expressed on D3, D7, and D14. The shared genes are indicated at the intersections of the circles in the Venn diagram. (C) GO analysis of the shared upregulated genes. (D) Venn diagram of downregulated genes that were significantly differentially expressed on D3, D7, and D14. (E) GO analysis of the shared downregulated genes.

Our analysis of the RNA-seq data for Gencode-annotated mRNAs showed that 24,881 mRNAs were detected. To gain insight into the roles of these mRNAs, we performed comparative expression analyses according to the differentiation stages of the osteoblasts (D3 vs. D0, D7 vs. D0, and D14 vs. D0). As shown in Fig. 2A, 664 mRNAs were upregulated and 477 mRNAs were downregulated on D3. On D7, 969 mRNAs were upregulated and 485 mRNAs were downregulated. On D14, 1,046 mRNAs were upregulated and 732 mRNAs were downregulated. A Venn analysis showed that 290 genes were simultaneously upregulated on D3, D7, and D14 (Fig. 2B). These genes were enriched in bone remodeling-associated processes, including ECM organization and focal adhesion. Genes associated with cell differentiation and cell apoptosis were also upregulated (Fig. 2C). In contrast, 194 genes were downregulated on D3, D7, and D14 (Fig. 2D). These genes were enriched mainly in processes related to the regulation of cellular proliferation, including mitotic nuclear division and sister chromatid cohesion (Fig. 2E).

### Characterization of lncRNA transcriptomes

**Figure 3:**
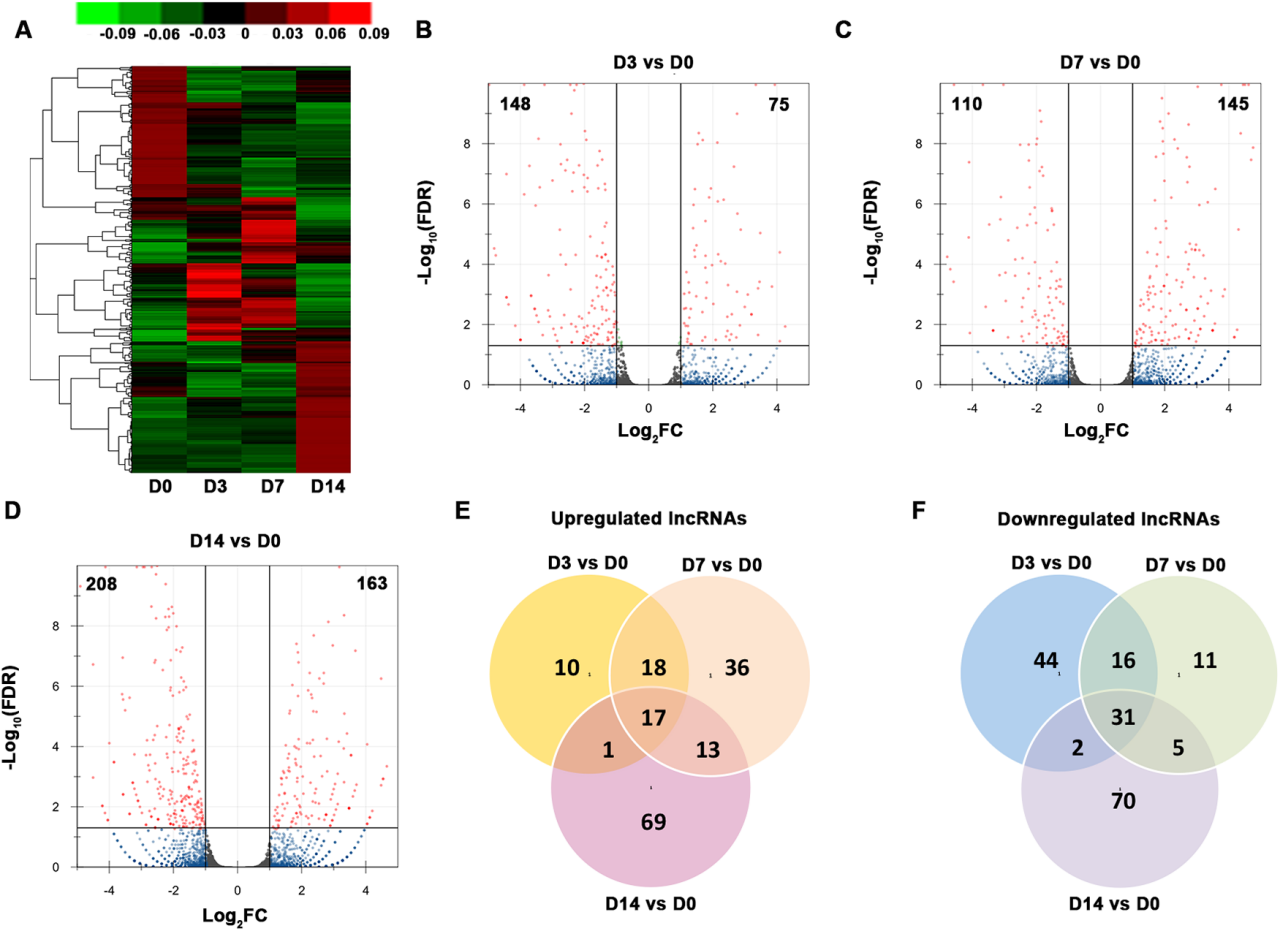
Characterization of the lncRNA transcriptome. (A) Overall profiles of lncRNAs in PDLSCs. Volcano plots were constructed to demonstrate the fold change (*x*-axis) and false discovery rate (*y*-axis) of lncRNAs that were differentially expressed on D3 (B), D7 (C), and D14 (D). (E) Venn diagram of the significantly upregulated lncRNAs on D3, D7, and D14. (F) Venn diagram of the significantly downregulated lncRNAs on D3, D7, and D14.

We organized the lncRNAs according to their expression profiles. The overall lncRNA expression profiles are presented in Fig. 3A. Small distinct groups of lncRNAs were expressed either early or late in osteogenic differentiation or predominately at one specific time point (e.g., D3 or D7). A much larger group was expressed mainly on D0 and D14. A pairwise comparison of lncRNA expression patterns during the osteogenic differentiation revealed that, from D0 to D3, 75 genes were upregulated and 148 genes were downregulated (Fig. 3B). Compared with the PDLSCs on D0, 145 genes were upregulated and 110 genes were downregulated on D7 (Fig. 3C), while 163 upregulated genes and 208 downregulated genes were detected on D14 (Fig. 3D). A Venn diagram showed that 17 genes were simultaneously upregulated and 31 lncRNAs were downregulated on D3, D7, and D14 (Figs. 3E and 3F).

### Temporal gene expression patterns of mRNAs

**Figure 4:**
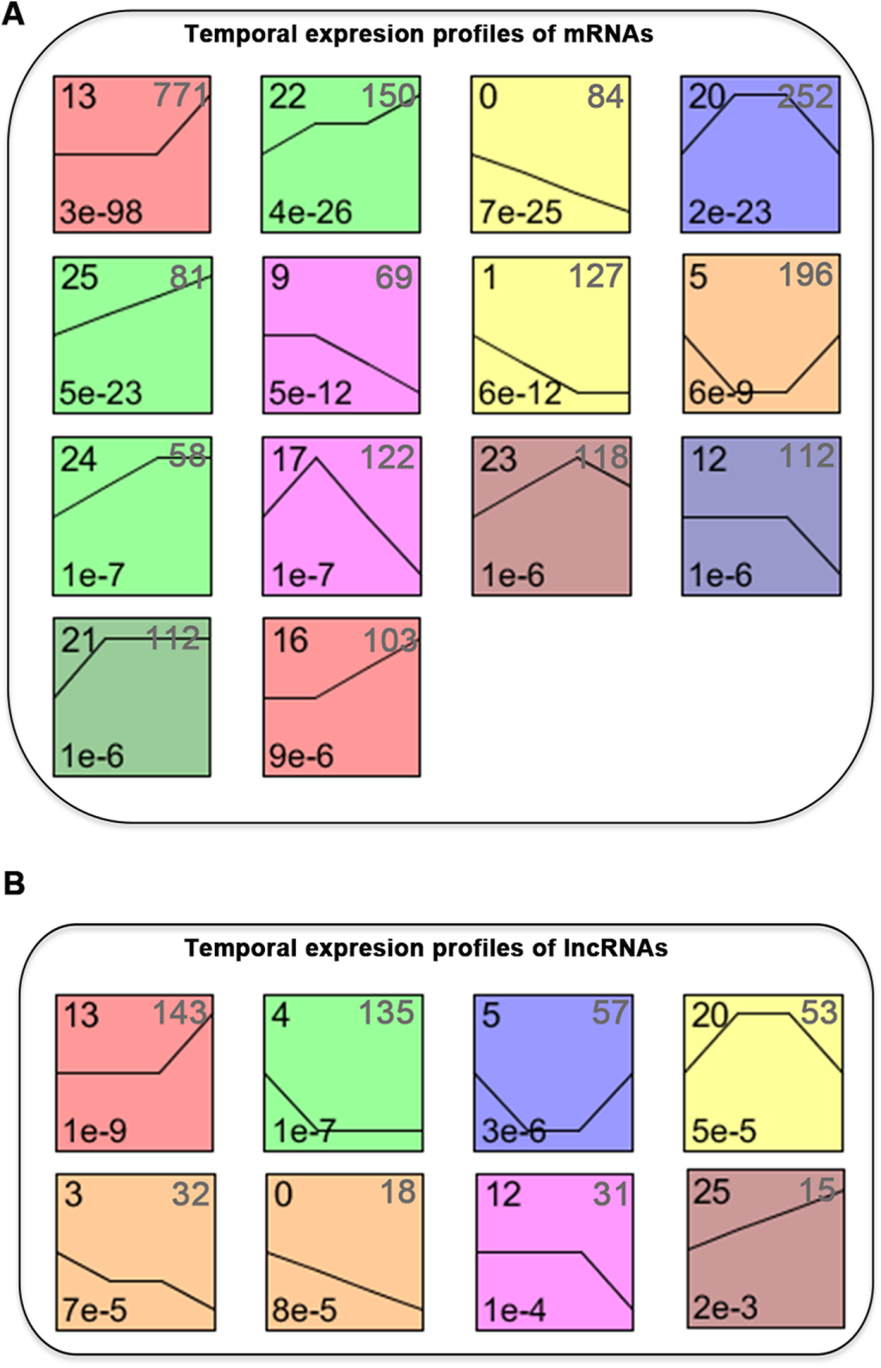
STEM identified the temporal expression profiles of mRNAs (A) and lncRNAs (B) with a *p*-value < 0.05. The profile number on the top left corner of each profile box was assigned by STEM, the number on the bottom left represents the adjusted *p*-value, and the number on the top right corner represents the cardinality of each cluster.

**Figure 5:**
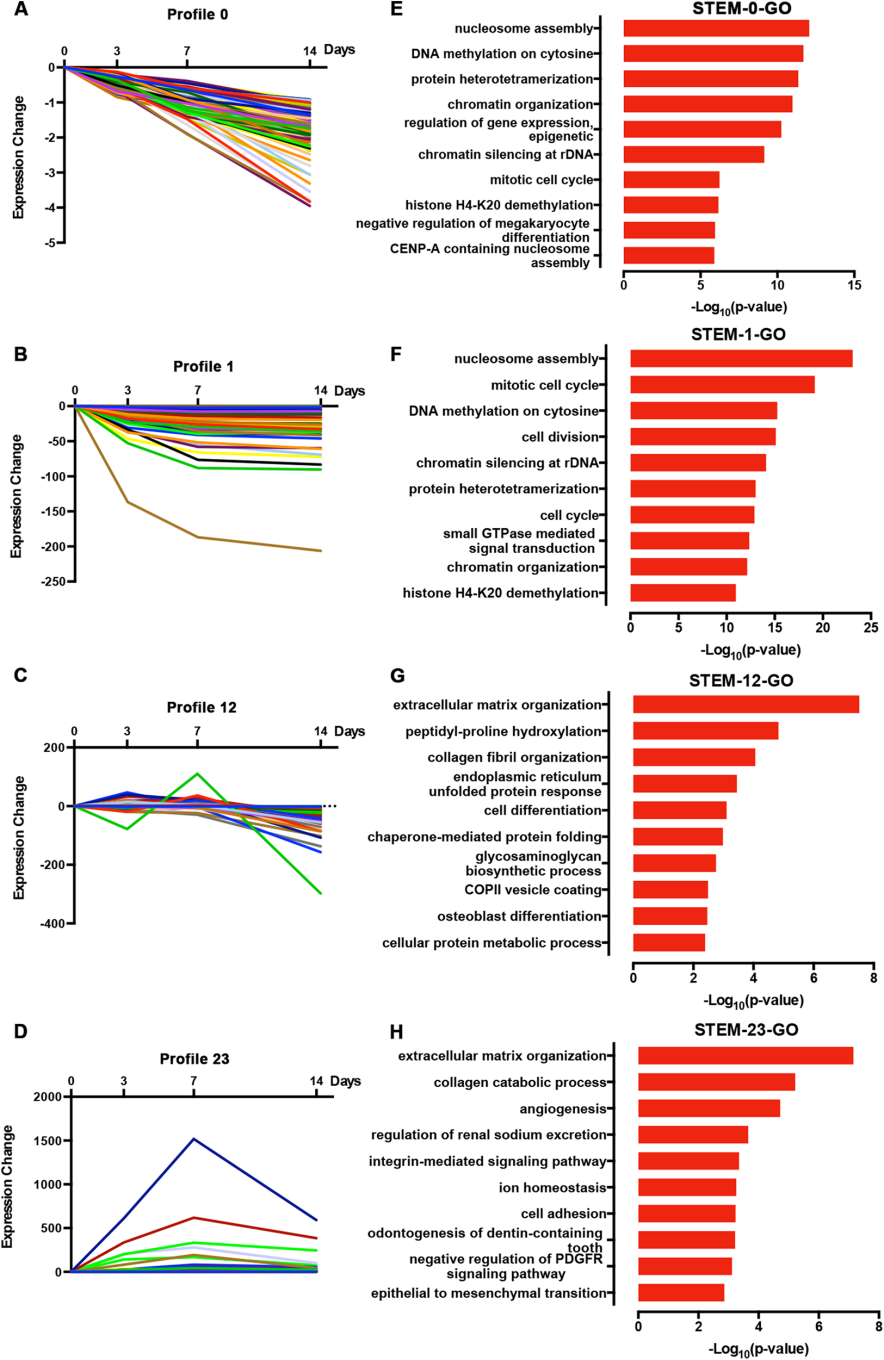
Expression patterns of profile 0 (A), 1 (B), 12 (C), and 23 (D) and GO analysis of genes clustered in 0 (E), 1 (F), 12 (G), and 23 (H).

We normalized the sequencing data to D0 and identified the temporal gene expression profiles using STEM. Within the 25 model profiles, 14 temporal mRNA profiles were statistically significant (Fig. 4A). The black lines in the profile boxes depict the gene expression patterns over the four time points. The profile number on the top left corner of each profile box was assigned by STEM, the number on the bottom left represents the adjusted *p*-value, and the number on the top right corner represents the cardinality of each cluster. We found continuous upregulation and downregulation patterns in profiles 0 (Fig. 5A) and 25. Gradual increases or decreases were distributed across profiles 1 (Fig. 5B), 9, 12 (Fig. 5C), 13, 16, 21, 22, and 24. Meanwhile, profiles 5, 17, 20, and 23 (Fig. 5D) showed biphasic responding expression patterns. Genes in profiles 0, 1, 12, and 23 were summarized in File S1. We then analyzed the genes associated with these profiles using GSEA analysis and retrieved the enriched GO terms.

Among the 14 profiles, the genes in profile 0 were associated with epigenetic regulation, including DNA methylation and histone modification (Fig. 5E). Profile 1 genes were enriched in cell mitosis (Fig. 5F). Profile 12 genes were associated with cell differentiation and osteoblast differentiation (Fig. 5G), while profile 23 genes were enriched in the collagen catabolic process, odontogenesis, and skeletal system development (Fig. 5H; *p*-value = 0.003). We found that 17 out of 112 genes in profile 12 were annotated as cell differentiation and osteoblast differentiation, and eight out of 118 genes in profile 23 were annotated as odontogenesis and skeletal system development. Specifically, COL6A1, VCAN, RRBP1, and CREB3L1 were annotated as osteoblast differentiation. Genes annotated as odontogenesis and skeletal system development included AMELX, BMP2, LAMA5, SCN5A, COL3A1, BMP1, ANKH, and COL12A1.

**Figure 6:**
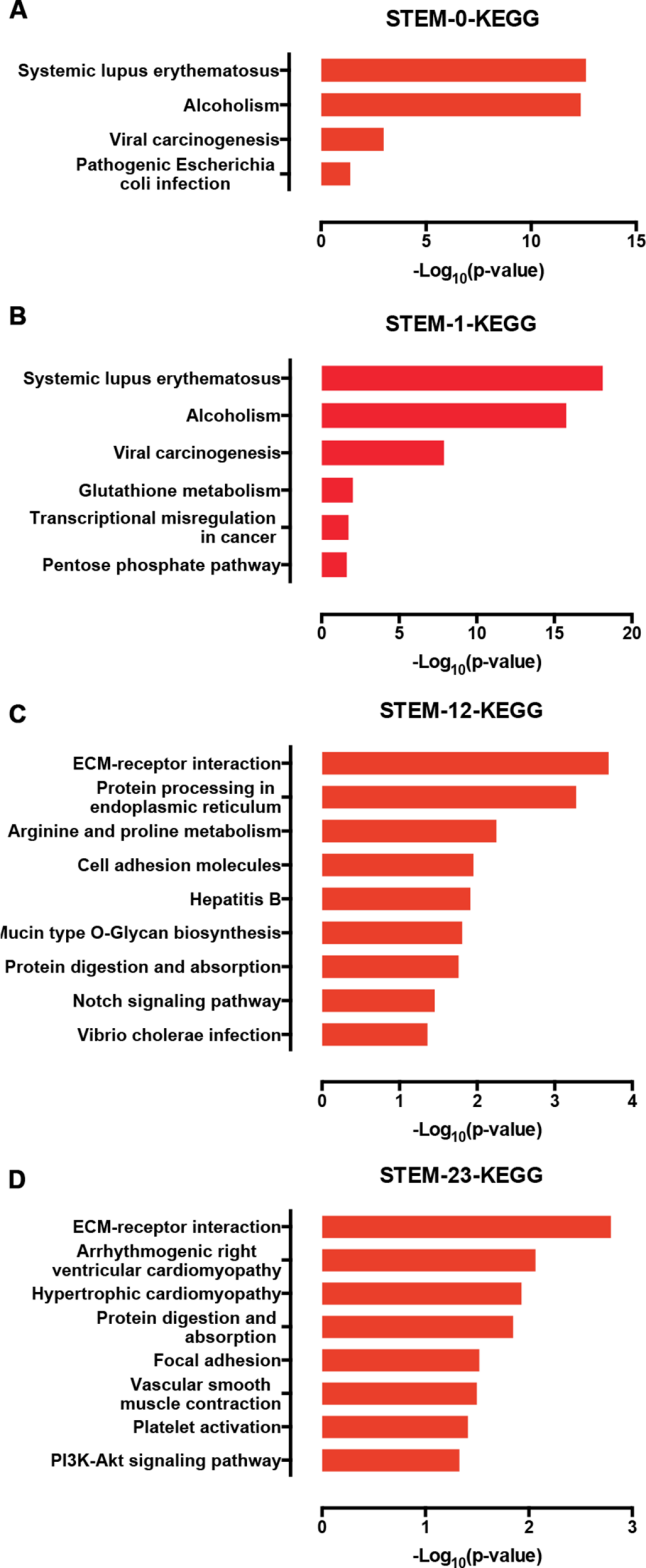
The KEGG signaling analysis of genes in the profiles 0 (A), 1 (B), 12 (C), and 23 (D).

Furthermore, we used a KEGG pathway analysis to explore the signaling pathways of DEGs involved in profiles 0, 1, 12, and 23. Interestingly, the genes in profile 0 and profile 1 were enriched in signaling associated with cancer development (Figs. 6A and 6B), which is characterized by the misregulation of cell proliferation and apoptosis. Genes in the profile 12 and 23 were not only enriched in ECM-receptor interactions, but also in the Notch and PI3K-AKT signaling pathways (Figs. 6C and 6D).

### Temporal gene expression patterns of lncRNAs

We classified the differentially expressed lncRNAs into 25 profiles according to the STEM analysis results and ranked the profiles according to their significance (*p*-value). The *p*-values of 8 clusters were statistically significant (Fig. 4B). The lncRNAs in clusters 0 and 25 represented lncRNAs whose expression was continuously increased or decreased. The lncRNAs in clusters 12 and 13 revealed differential expression at D7, while lncRNAs in clusters 16 and 13 revealed differential expression at both D7 and D14. lncRNAs in clusters 5, 17, 20, and 23 corresponded to lncRNAs with biphasic expression patterns. Since the differentially expressed mRNAs in gene expression profile 12 were significantly enriched in osteoblast differentiation, the lncRNAs included in profile 12 were potentially related to osteogenic differentiation of PDLSCs.

### Validation of the expression of mRNAs and lncRNAs that associated with osteoblast differentiation

**Figure 7:**
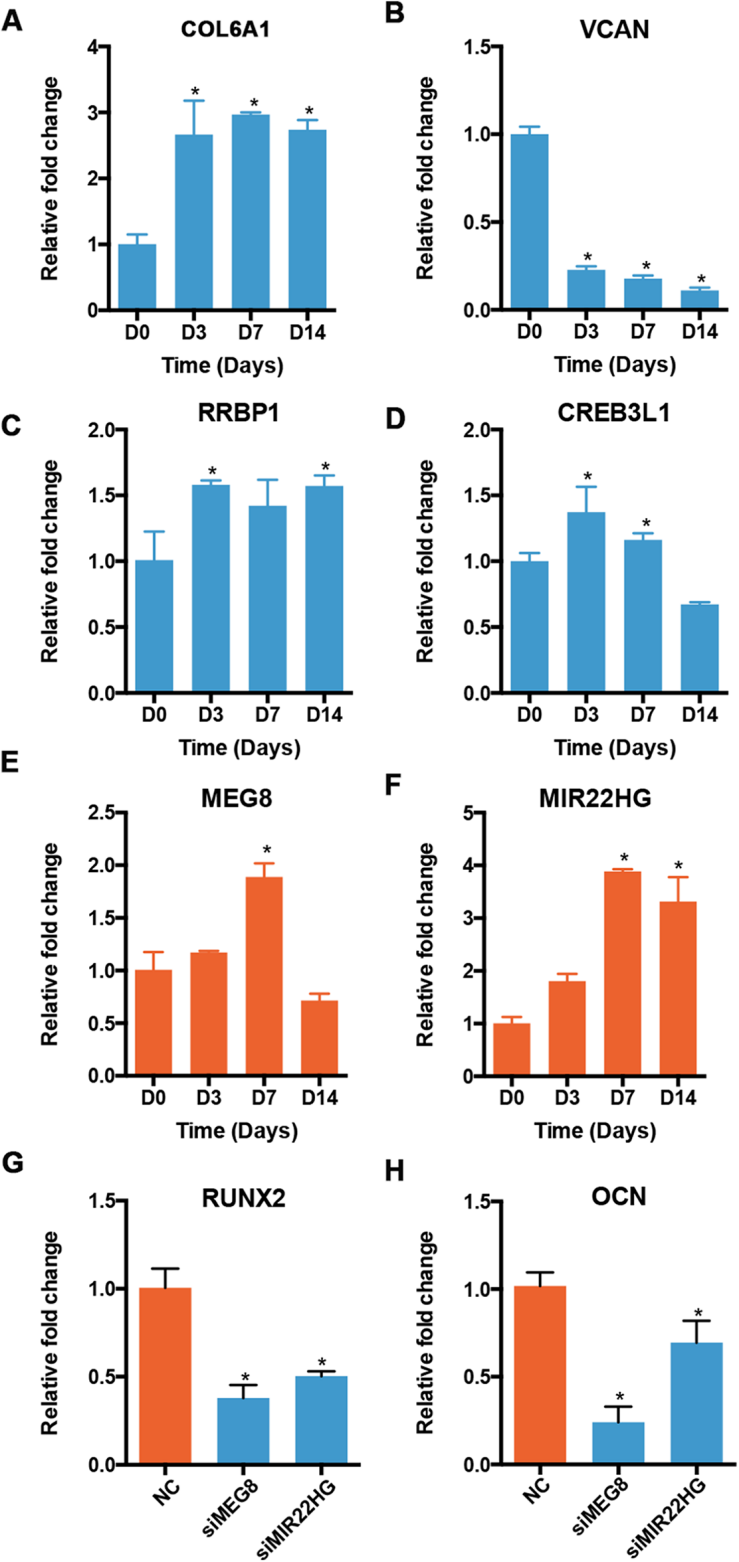
Validation of the expression of genes in profile 12. The expression of mRNAs, including COL6A1 (A), VCAN (B), RRBP1 (C), and CREB3L1 (D), and lncRNAs, including MEG8 (E) and MIR22HG (F), was analyzed using qRT-PCR. The expression of RUNX2 (G) and OCN (H) was also analyzed after MEG8 and MIR22HG were knocked down. *p < 0.05 compared to D0.

We validated the expression of osteogenic mRNAs and lncRNAs in the profile 12 using qRT-PCR. Though most lncRNAs were expressed at a low level, some lncRNAs were highly expressed, rivaling the expression of many mRNAs. Therefore, we applied an expression cutoff to remove the transcripts that were expressed at a low level. We found that two lncRNA genes in the profile 12 were expressed at a RPKM of more than 10. The expression patterns of mRNAs and lncRNAs showed similar trends. The levels of COL6A1 and RRBP1 were increased at D3 and stayed relatively stable afterward (Figs. 7A and 7C). The expression of VCAN was decreased significantly during the osteoblast differentiation of PDLSCs (Fig. 7B). CREB3L1 increased significantly at D3, but decreased from D7 to D14 (Fig. 7D). For the lncRNAs, the expression of MEG8 was significantly increased at D7, but reduced at D14 (Fig. 7E). Overall, the expression of MIR22HG was upregulated in the osteogenic medium, and its level peaked at D7 (Fig. 7F). Moreover, the expression of RUNX2 and OCN decreased once the siRNA targeting MEG8 and MIR22HG were applied to PDLSCs, indicating an osteogenic role for the two lncRNAs (Figs. 7G and 7H).

## Discussion

Utilizing PDLSCs to regenerate periodontal structures is a promising method for functional periodontal tissue regeneration and bone regeneration (Liu et al., 2008; Sonoyama et al., 2006). A complex network of signaling molecules regulates the differentiation of MSCs like PDLSCs into osteoblasts (Chen et al., 2016a; Lin & Hankenson, 2011). lncRNAs have been found to regulate mRNA expression levels and maintain normal biological function. Studies suggest that lncRNAs are also involved in the osteogenic differentiation of PDLSCs (Jia, Jiang & Ni, 2015; Wang et al., 2016). The discovery of this regulatory mechanism has expanded our understanding of biological processes and organism complexity. Recently, the lncRNA expression profile was examined after 21 days of culturing the PDLSCs in osteogenic medium using microarray analysis (Qu et al., 2016). However, since the dynamics of gene expression are characterized by a phasic pattern, the expression profiles of genes at a single time point are insufficient to fully characterize the role of lncRNAs in the osteogenic differentiation of PDLSCs. In the present study, we therefore aimed to identify molecular events governing the differentiation of PDLSCs to osteoblasts, using STEM to assess the expression profiles of lncRNAs and mRNAs.

A comparison of the mRNA transcriptional profiles of PDLSCs on D0 and at later time points, along with a GO analysis, revealed that genes that allowed ECM organization and focal adhesion were upregulated. In contrast, genes that promoted cell proliferation were downregulated. During osteoblastogenesis, the differentiation of stem cells can be subdivided into several stages, including cellular proliferation and differentiation, and ECM synthesis, maturation, and mineralization. Each stage is characterized by changes in gene expression patterns. Early-stage osteoblasts predominantly support proliferation and ECM biosynthesis, while late-stage osteoblasts mediate gene expression for ECM maturation and mineralization (Karner et al., 2009). During the late stages of differentiation, cell-to-cell contact at high densities inhibits proliferative activity and triggers stem cell differentiation. Accordingly, the expression of many osteoblast-specific genes, such as osterix and OCN, is upregulated at the end of active proliferation. In our study, we also found that the inhibition of active proliferation was associated with a terminally differentiated osteoblastic phenotype.

We also used a STEM platform to investigate how gene expression profiles change continuously over time during the osteoblast differentiation of PDLSCs. We selected 25 predetermined temporal model profiles and determined the number of genes assigned to each profile. Some distinct gene expression patterns were noted as significant during osteoblast differentiation. For example, profile 0 genes that modulate epigenetic regulation, such as EZH2 and ERCC2, were constitutively downregulated. Epigenetic regulation represents as a link between genetic aspects and environmental influences, and it is involved in bone biology and osteoblast differentiation (Pike et al., 2015). Activation of EZH2 promotes osteogenic differentiation of MSCs via histone modification (Wei et al., 2011; Chen et al., 2016b); and mutations in Ercc2 result in decreased bone volume and strength in mice (Nicolaije et al., 2012). Genes in profile 1, like NUF2, were transiently induced at early stages and were associated with cell cycle and mitosis. In agreement with our results, the silencing of NUF2 was shown to lead to cell-cycle arrest (Fu & Shao, 2016).

On the other hand, genes in profile 12 showed a dip pattern from D7 to D14 and were associated with osteoblast differentiation. Additionally, the expression of genes in profile 23 that were associated with mineralization peaked at D7 and dipped to a lower level at D14. Specifically, we found that BMP1 and BMP2 belong to the profiles 12 and 23 (Fig. S2). BMP1 is an astacin metalloprotease that processes ECM proteins (Asharani et al., 2012; Ge & Greenspan, 2006), and BMP2 is recognized as the strongest osteoinductive molecule inducing the formation of the mineralized matrix (Costello et al., 2015). Moreover, we identified the osteoblast-associated genes in profile 12 and validated the expression of COL6A1, VCAN, CREB3L1, and RRBP1 using qRT-PCR. Consistently, these four genes were previously shown to be involved in osteoblastogenesis. The protein of COL6A1 is a constituent for microfibril and is involved in cell differentiation and embryonic development (Frka et al., 2009). Genetic deletion of Col6a1 results in bone mass reduction and bone fragility (Izu et al., 2016). VCAN, also known as versican, is an abundantly expressed proteoglycan in the ECM compartment, and the expression of VCAN is enhanced by RUNX2 (Teplyuk et al., 2009). RUNX2 is critical for the commitment to osteoblast lineage, and global deletion of RUNX2 leads to an absence of osteoblasts and failed ossification (Ducy et al., 1997; Komori et al., 1997). These findings indicate that VCAN is essential for bone matrix deposition. CREB3L1 is an endoplasmic reticulum-resident transcription factor involved in angiogenesis and osteoblast differentiation (Chen et al., 2014; Cui et al., 2015).

We also identified two lncRNAs that might be correlated with osteoblast differentiation using the STEM algorithm. MEG8, similar as MEG3, is a maternally imprinted gene in the Dlk1-Dio3 locus and is highly expressed in diverse organs of mouse embryos (Gu et al., 2012). In the present study, we found that the expression profiles of MEG3 and MEG8 were similar. MEG3 was found to promote osteoblast differentiation of MSCs and to inhibit adipogenesis via miR-140-5p (Li et al., 2017; Zhuang et al., 2015). Although a role for MEG8 in osteoblast differentiation has not been reported, the MEG8 locus is hypermethylated in the patients with a recognizable phenotype including premature puberty and a short stature (Beygo et al., 2017). These results indicate that MEG8 may be involved in skeletal development and osteogenesis. MIR22HG is transcribed from the gene locus harboring miR-22 and inhibits cell invasion in hepatocellular carcinoma by deriving miR-22 (Liu, Zhang & Wu, 2016). Recently, MIR22HG was found to inhibit cell proliferation by targeting miR-141-3p and upregulating DAPK1 expression (Cui et al., 2018). Since miR-141-3p inhibits osteoblast differentiation of MSCs (Qiu & Kassem, 2014), MIR22HG may also regulate osteoblast differentiation with a similar mechanism.

## Conclusion

In summary, in this study we characterized the mRNA and lncRNA transcriptomes of PDLSCs during the osteogenic differentiation. We used STEM to define the temporal patterns of mRNAs and lncRNAs in osteoblastogenesis, and we also identified two highly expressed lncRNAs, MEG8, and MIR22HG, that play a potential role in osteoblast differentiation. Our findings can be used to enhance the current understanding of the molecular events regulating osteoblast differentiation and can serve as a foundation for the identification of functional lncRNAs in the differentiation of PDLSCs.

## Supplemental Information

10.7717/peerj.5214/supp-1 Supplemental Information 1 Figure S1. The expression of BMP1 and BMP2.

10.7717/peerj.5214/supp-2 Supplemental Information 2 Figure S2. The quality control of the sequencing data.

10.7717/peerj.5214/supp-3 Supplemental Information 3 Genes in profiles 0, 1, 12, and 23.

10.7717/peerj.5214/supp-4 Supplemental Information 4 The differentially expressed genes at D3, D7, and D14.

## Additional Information and Declarations

### Data Availability

The following information was supplied regarding data availability: Raw data of the performed RNA sequencing have been recorded in the GEO public database (accession number: GSE99958).
