# Supplementary figures and images for: Time series clustering of mRNA and lncRNA expression during osteogenic differentiation of periodontal ligament stem cells
Source: PeerJ. 2018 Jul 16;6:e5214. doi: 10.7717/peerj.5214 (PMC6052852; doi:10.7717/peerj.5214)

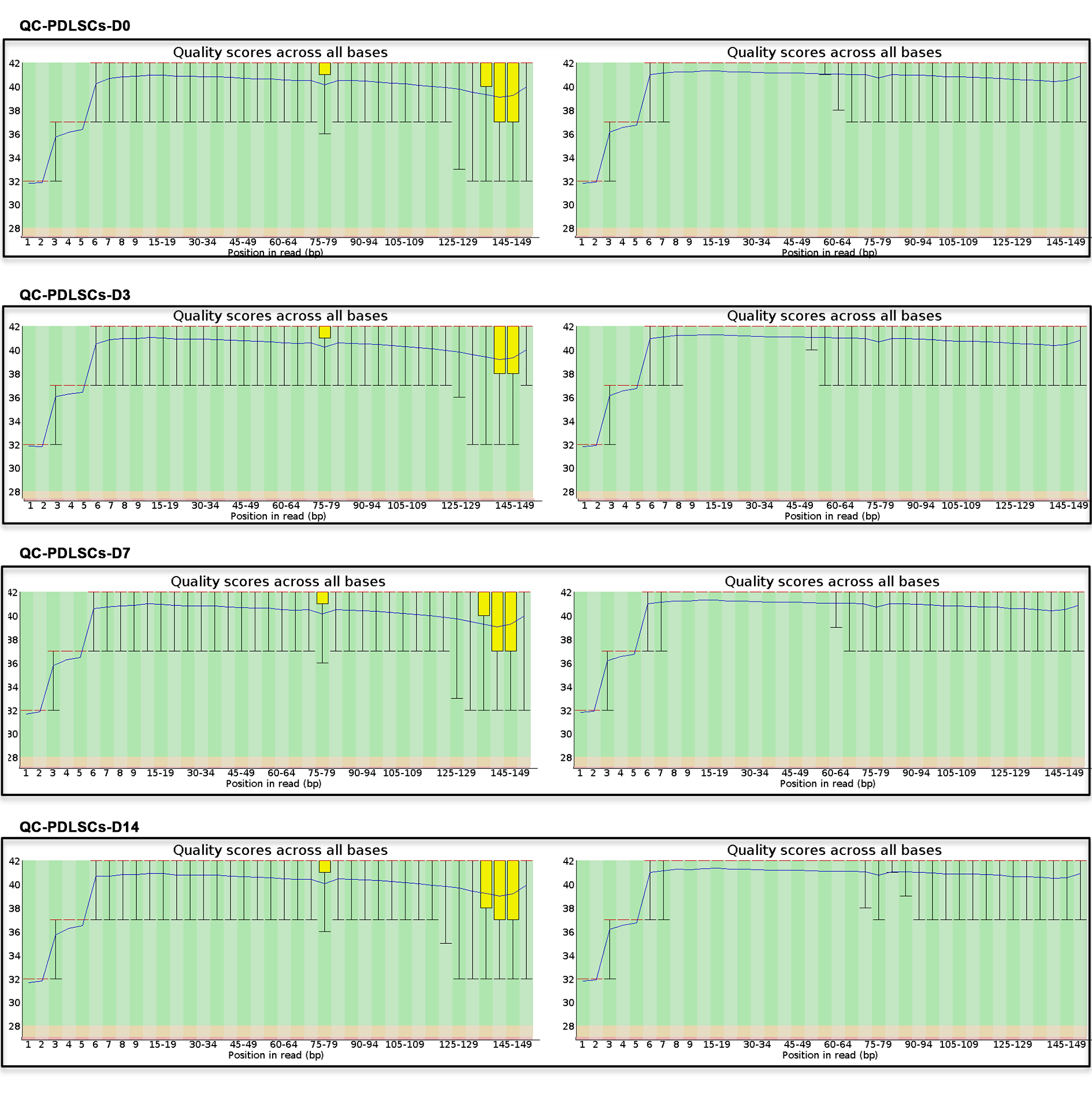

Supplement: Supplemental Information 1 [file peerj-06-5214-s001.png]

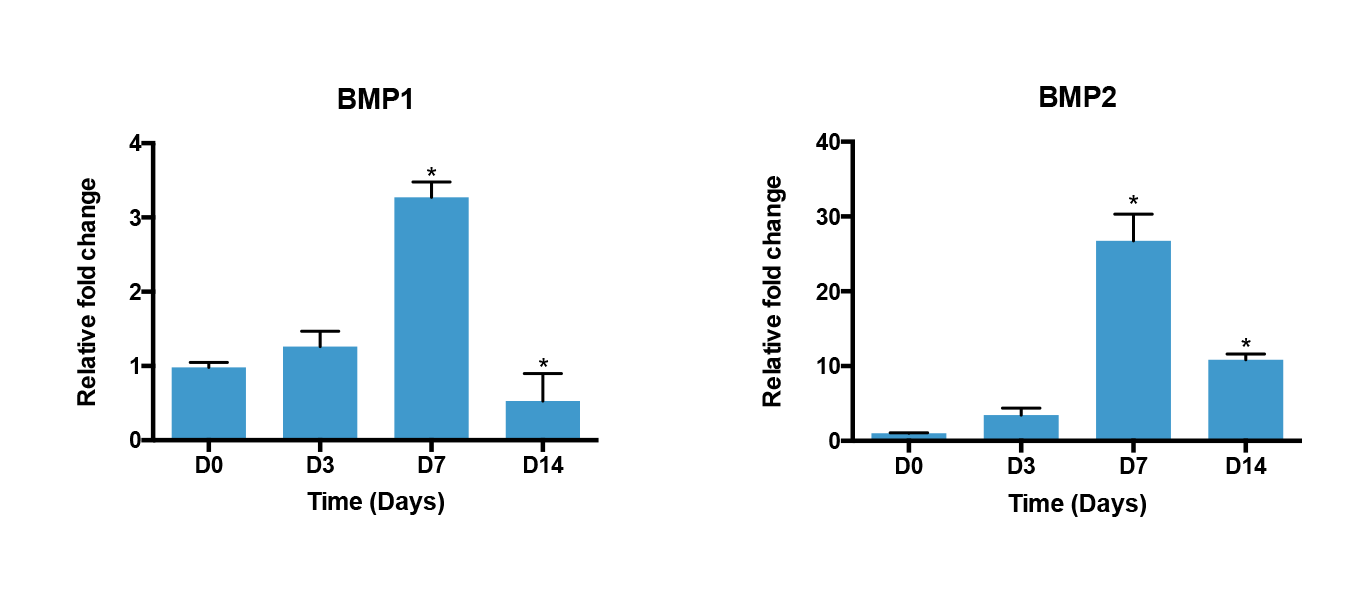

Supplement: Supplemental Information 2 [file peerj-06-5214-s002.png]
